# The complete mitochondrial genome of *Ripeacma umbellata* Wang, 2009 (Lepidoptera: Autostichidae)

**DOI:** 10.1080/23802359.2021.1920864

**Published:** 2021-06-14

**Authors:** Yan Zhi, Aihui Yin

**Affiliations:** a Morphological Laboratory, Guizhou University of Traditional Chinese Medicine, Guiyang, PR China; b Laboratory Animal Center, Guizhou Medical University, Guiyang, PR China

**Keywords:** *Ripeacma umbellata*, mitochondrial genome, autostichidae, phylogenetic analysis

## Abstract

The mitogenome of *Ripeacma umbellata* Wang, 2009 was reported in this study. It was 15,486 bps long and strongly AT biased, consisting of 13 protein-coding genes (PCGs), 22 transfer RNAs (tRNAs), 2 ribosomal RNAs (rRNAs), and 1 non-coding control region (351 bp). Most PCGs used the conventional ATN start codon, except for *cox1* and *cox2*. Four genes used single T residue as stop codon rather than the routinely used TAA or TAG. All tRNAs, except for TrnS1, could fold into the cloverleaf secondary structure. Bayesian inference phylogenetic tree built on 13 PCGs from *R. umbellata* and another 21 species in Gelechioidea demonstrated that genus *Ripeacma* was a member in Autostichidae, which was consistent with the latest phylogenetic study.

The small moth *Ripeacma umbellata* Wang, 2009 belongs to the genus *Ripeacma* Moriuti, Saito & Lewvanich (Autostichidae, Lepidoptera), which currently contains 34 species in Palearctic and Oriental regions (Li and Wang 2017; Kim and Lee 2017). The systematic position of *Ripeacma* within the superfamily Gelechioidea had long been a problem to all the involved researchers (e.g. Lvovsky 2005, 2015; Heikkilä et al. 2014; Kim and Lee 2017), until a recent molecular study seemed to have given it a solid answer, that *Ripeacma* in nature belonged to the family Autostichidae (Wang and Li 2020). In attempt to test this scientific assertion, we herein sequenced for the first time the mitogenome of a *Ripeacma* member, *R. umbellata* and used it as the representative to recover the phylogenetic relationship of this genus in Gelechioidea. The moths were collected from Maolan Natural Reserve (25°17′10″N, 108°42′42″E), Guizhou, China in 2020, using light trap. The specimens were then stored in absolute alcohol under −20 °C in the Morphological Laboratory of Guizhou University of Traditional Chinese Medicine, Guiyang, China (Aihui Yin, keyanlaodong@163.com) under the voucher number GZUTCM:M24–27.

The next-generation sequencing (NGS) was performed using Illumina HiSeq2500 platform in Sangon Biotech (Shanghai) Co., Ltd., China. The genome assembly and gap-filling were carried out with SPAdes version 3.14.1 (https://github.com/ablab/spades) (Bankevich et al. 2012), ARC version 1.1.3 (https://github.com/ibest/ARC) (Hunter et al. 2015), BWA version 0.7.17 (https://sourceforge.net/projects/bio-bwa/) (Li 2013), and samtools version 0.1.19 (https://github.com/samtools/samtools) (Li et al. 2009). Sequence polish was aided with Pilon version 1.23 (https://github.com/broadinstitute/pilon) (Walker et al. 2014). MITOS WebServer (http://mitos2.bioinf.uni-leipzig.de/index.py) was utilized for annotation.

The 15,486 bp long circular mitogenome of *R. umbellata* (GenBank: MW366997) was constructed. It was strongly AT biased (AT 79.9%, CG 20.1%), and shared the typical set of genes (13 protein-coding genes [PCGs], 22 transfer RNAs [tRNAs], and 2 ribosomal RNAs [rRNAs]) with other metazoan animals (Wolstenholme 1992). Most PCGs of *R. umbellata* used typical start codon ATN at initiation, only *cox1* and *cox2* started with unorthodox codons CGA and TTG respectively. In terms of stop codon, most PCGs used the routine TAA or TAG, except for *cox1, cox2, nad4*, and *nad5*, which used a single T residue to stop transcription. All tRNAs could fold into the clover-leaf secondary structure, excluding TrnS1, due to its lack of the dihydrouracil arm. The special tRNA gene order TrnM-TrnI-TrnQ which was shared among mitogenomes of almost all Ditrysian moths was also observed in *R. umbellata* (Cao et al. 2012; Park et al. 2016). The putative A + T rich control region was 351 bps in length, and had very high AT ratio (93.2%).

MrBayes version 3.2.7 (http://nbisweden.github.io/MrBayes/sourseforge) (Ronquist et al. 2012) was run for the data set built of 13 PCGs from *R. umbellata* plus another 21 species in Gelechioidea to generate a BI phylogenetic tree using ‘GTR + I + G’ substitution model (Figure 1). The families Autostichidae, Gelechiidae, Stathmopodidae, and Xyloryctidae that had multiple representatives were successfully recovered as monophyletic. However, Oecophoridae appeared to be polyphyletic in this study. *R. umbellata* was indeed a member of the family Autostichidae as illustrated by Wang and Li (2020).

**Figure 1.**
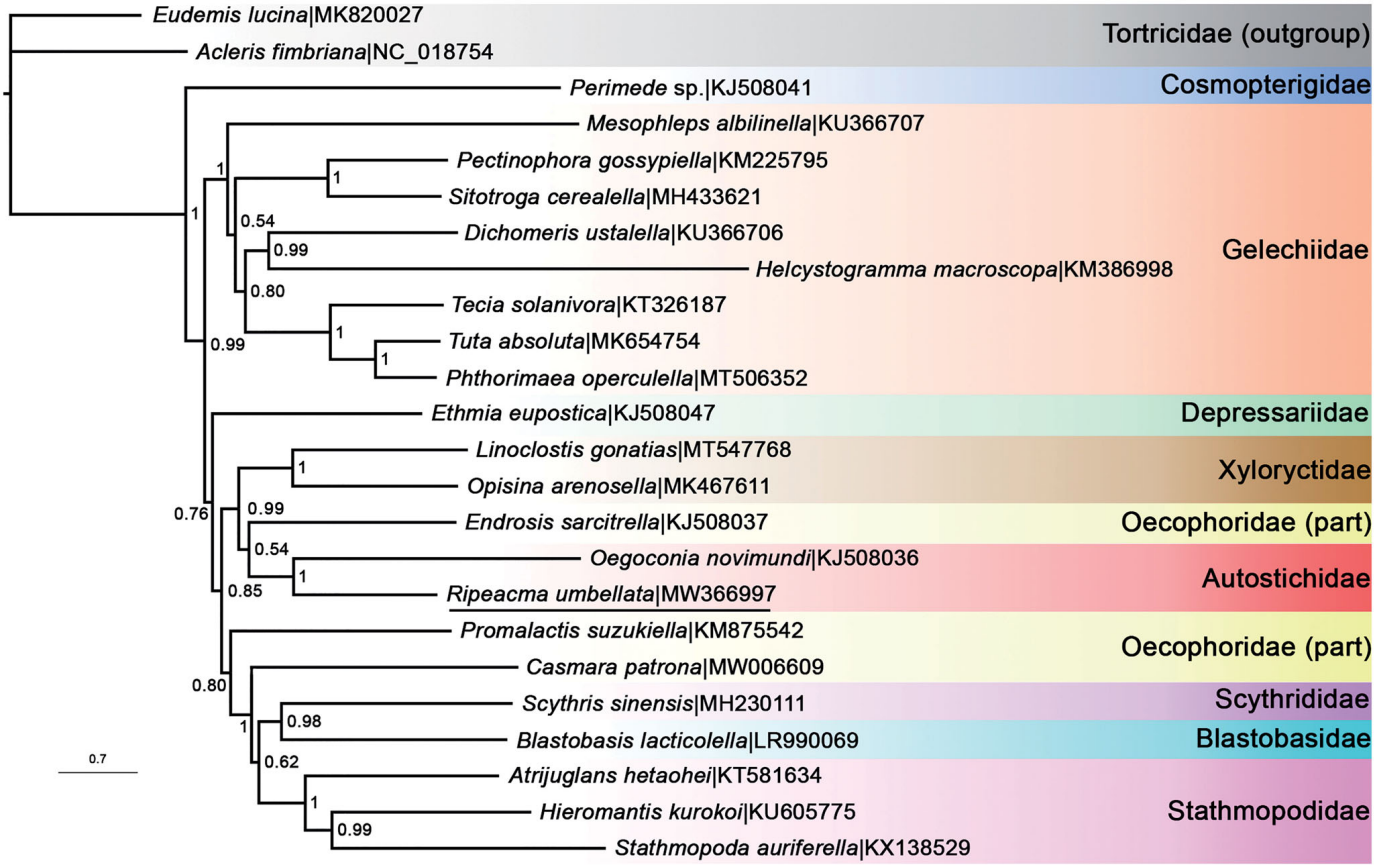
BI tree showed phylogenetic relationships within superfamily Gelechioidea. Bayesian posterior probabilities were labeled at nodes and GenBank accession numbers were indicated in the tree (*R. umbellata* was underlined). Two representatives from Tortricidae were set as outgroup.

## Data Availability

The genome sequence data that support the findings of this study are openly available in GenBank of NCBI at https://www.ncbi.nlm.nih.gov/nuccore/MW366997 under the accession no. MW366997.
